# Impact of Arterial Reconstruction With Recipient's Own Internal Iliac Artery for Multiple Graft Arteries on Living Donor Kidney Transplantation: Strobe Study

**DOI:** 10.1097/MD.0000000000001811

**Published:** 2015-10-30

**Authors:** Takahisa Hiramitsu, Kenta Futamura, Manabu Okada, Takayuki Yamamoto, Makoto Tsujita, Norihiko Goto, Shunji Narumi, Yoshihiko Watarai, Takaaki Kobayashi

**Affiliations:** From the Department of Transplant and Endocrine Surgery, Nagoya Daini Red Cross Hospital (TH, KF, MO, TY, MT, NG, SN, YW); and Department of Renal Transplant Surgery, Aichi Medical University School of Medicine, Aichi, Japan (TK).

## Abstract

The aim of this study is to investigate the usefulness of arterial reconstruction using the recipient's own internal iliac artery for multiple kidney graft arteries.

The safety and efficacy of various arterial reconstruction methods have been demonstrated. Although some reports have documented arterial reconstruction with the recipient's own internal iliac artery for multiple kidney graft arteries using the interposition method, usefulness of this technique has not yet been investigated compared with other arterial reconstruction methods.

Between January 2008 and April 2014, 532 living donor kidney transplants in adult recipients were performed at 1 center. Of these, 389 kidney grafts had a single artery and did not need arterial reconstruction (nonarterial reconstruction group). Among the bench surgery patients, 19 kidney grafts for multiple arteries were performed using the interposition method with the recipient's own internal iliac artery (interposition group). Seventy-nine kidney grafts were performed using conjoined reconstruction (conjoined group) and 15 kidney grafts were performed using end-to-side reconstruction (end-to-side group). Total ischemic time (the period between arterial clamp and blood reperfusion), time to initial urination, perioperative and postoperative estimated glomerular filtration rate (eGFR), and complication rates between the interposition group and other 3 groups were retrospectively investigated. This study was based on the STROBE compliant.

Warm ischemic time (the period between arterial clamp and beginning of the cold perfusion) of interposition group was significantly longer than that of nonarterial reconstruction group. Total ischemic time of the interposition group was significantly longer than those of other 3 groups. But time to initial urination, perioperative and postoperative eGFR, and complications were similar to other 3 groups.

The interposition method was shown to be a useful standard method for multiple kidney graft arteries of living donor kidney transplantation in carefully selected recipients without calcification of the iliac arteries.

## INTRODUCTION

The first laparoscopic donor nephrectomy was reported by Ratner et al in 1995.^1^ Wolf et al introduced hand-assisted laparoscopic donor nephrectomy in 1998.^2^ Since then, laparoscopic procedures have been widely performed for living donors. Previously, some reports did not recommend laparoscopic procedures for the donors of multiple graft arteries due to the frequent complications.^3,4^ Nevertheless, now several reports have addressed the operative indications of laparoscopic procedures for the donors of multiple graft arteries.^5–7^ However, graft arterial length is shorter with laparoscopic procedures than with open procedures because of the wide endostaple lines. Kidney grafts with early branched arteries often lead to multiple arteries after the removal of staples. For these multiple arteries, arterial reconstructions were necessary. Many techniques of arterial reconstruction have been reported, such as the conjoined method, the end-to-side method, and the method involving the inferior epigastric artery. The efficacy and safety of these methods has also been reported before.^8^ Only the operative technique of arterial reconstruction with the recipient's own internal iliac artery for multiple arteries (interposition method) has likewise been reported,^9–12^ but the usefulness of this method compared with that of a single arterial graft, conjoined method, and end-to-side method has not yet been demonstrated. In this study, we investigated the usefulness of interposition methods compared with that of a single arterial graft (nonarterial reconstruction), conjoined method, and end-to-side method.

## METHODS

### Study Design

To investigate the usefulness of the interposition method, adult recipients of living donor kidney transplants were divided into 4 groups according to the operative methods used. One was the interposition group and the others were nonarterial reconstruction group, conjoined group, and end-to-side group. Usefulness between interposition group and other 3 groups was evaluated with perioperative and postoperative estimated glomerular filtration rate (eGFR), and complications.

### Participants

**FIGURE 1:**
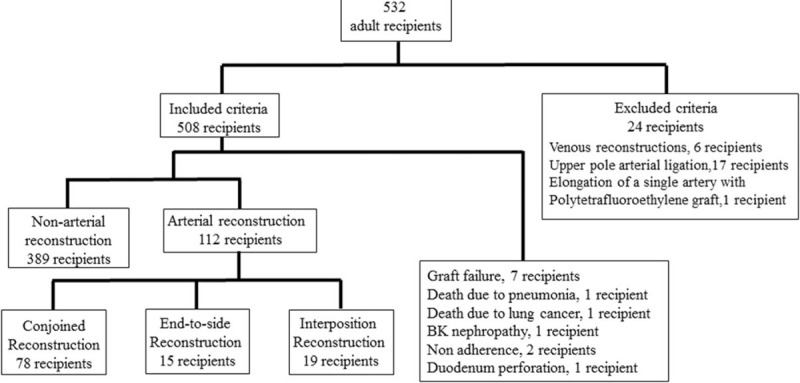
SRTOBE flow chart.

Between January 2008 and April 2014, 532 consecutive living donor kidney transplants were performed in adult recipients at our hospital. Of these, 24 were excluded from this study due to the following reasons: 1 out of 24 underwent elongation of a single artery with polytetrafluoroethylene graft, 6 underwent venous reconstruction, and very thin arteries feeding the upper pole were ligated in 17. The remaining 508 recipients were enrolled in the study and observed every month from January 2008 to September 2014 (mean observation period: 41.9 ± 21.3 months) (Fig. 1). All patients’ data were retrospectively collected from patients’ charts without missing data.

### Ethical Review

This study was approved by the institutional review board in Nagoya Daini Red Cross Hospital.

### Outcome Measures

Total ischemic time (TIT) (the period between arterial clamp and blood reperfusion), time to initial urination (the period between blood reperfusion and urination from the graft ureter), perioperative and postoperative estimated glomerular filtration rate (eGFR), and complications were compared. Complications included arterial thrombosis, urine leakage, ureteric stricture, delayed graft function, postoperative bleeding, lymphocele, acute cellular rejection, and antibody-mediated rejection.

### Preoperative Evaluation and Indication of Interposition Method

Preoperative kidney functions and anatomical features were evaluated with Tc-99m diethylene-triamine-pentaacetate and enhanced CT images. Procurement side was determined according to kidney function and donor kidney problems, such as renal stones and arterial aneurysms, regardless of the number of renal arteries. The number of kidney graft arteries was evaluated preoperatively with enhanced 3D-CT images. The condition of the recipient's iliac artery was also evaluated with CT images. For the recipients without calcified iliac arteries, interposition method was selected. Conjoined method and end-to-side method were the first choice for arterial reconstruction, if the arterial length and distance between arteries were appropriate. However, if arterial conditions were not appropriate for conjoined method and end-to-side method, interposition method was then selected.

### Interposition Methods

**FIGURE 2:**
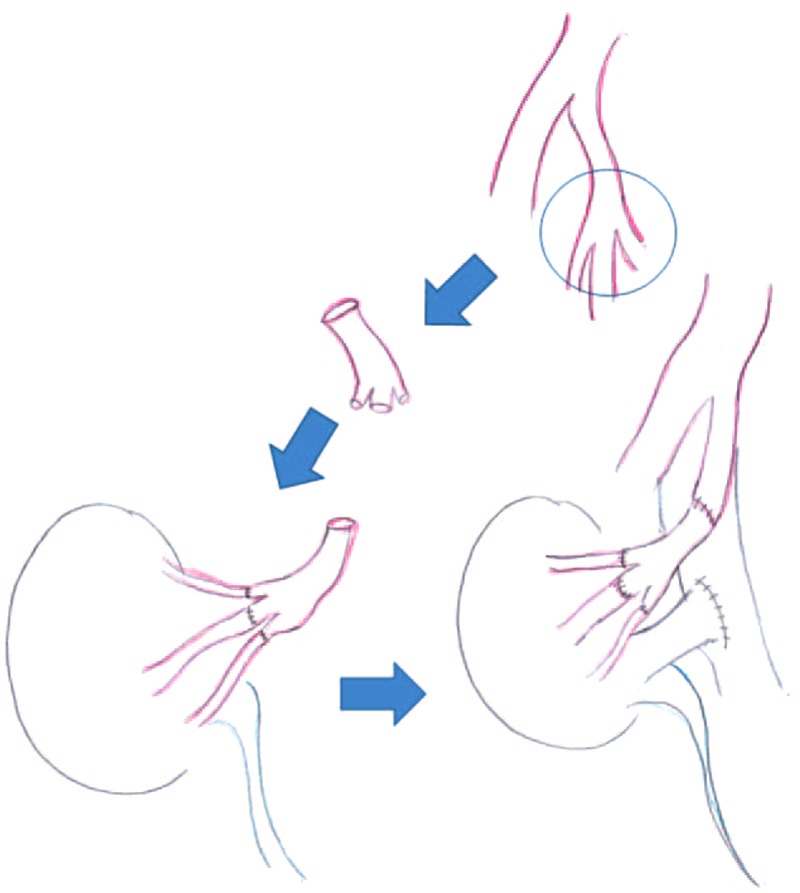
Operation technique of arterial reconstruction with recipient's internal iliac artery for multiple kidney graft arteries (interposition method).

Before bench surgery, the recipient's internal iliac artery was exposed to the peripheral branches of the anterior and posterior branch. After ligating the peripheral branches of the anterior and posterior branch, the internal iliac artery was clamped. The internal iliac artery was cut at the peripheral branches of the anterior and posterior branch and in the middle of the internal iliac artery. Next, the arterial graft was procured. In bench surgery, each artery of kidney graft was anastomozed end-to-end with each peripheral branch of the internal iliac artery. The kidney graft was then implanted by reanastomosis of the internal iliac artery (Fig. 2).

### Statistical Analysis

Statistical analyses were performed using the independent-samples *t* test for continuous data, and *χ*^2^ or Fisher exact test for the categorical variables; *P* < 0.05 was considered significant. Analyses were performed using SPSS for Windows version 13.0 (IBM, Chicago, IL) statistical software.

## RESULTS

### Participants

Five hundred eight recipients were enrolled in the study and observed every month from January 2008 to September 2014 (mean observation period: 41.9 ± 21.3 months). During the observation period, 7 patients dropped out for the following reasons: 2 with functioning grafts died due to lung cancer and pneumonia, 1 developed BK virus nephropathy, 2 experienced acute cellular rejections due to nonadherence, and 1 developed graft loss due to perioperative duodenal perforation. As a result, 501 of the 532 recipients were observed at our hospital; no patients dropped out during this period. Three hundred eighty-nine out of 501 kidney grafts had a single artery and did not need arterial reconstruction (nonarterial reconstruction group). The interposition method was performed in 19 kidney grafts (interposition group). Conjoined method (conjoined group) and end-to-side method (end-to-side group) were performed in 78 and 15 kidney grafts, respectively (Fig. 1).

### Descriptive Data

Characteristics of recipients, donors, and kidney grafts for the interposition group (19 recipients), conjoined group (78 recipients), end-to-side group (15 recipients) and the nonarterial reconstruction group (389 recipients) were shown in Table 1. There was no significant difference between interposition group and other 3 groups except for the number of arteries (Table 2). Kidney grafts with more than 2 arteries were significantly more in interposition group than those of other 3 groups. All patients’ data collected were complete and accurate.

**TABLE 1:**
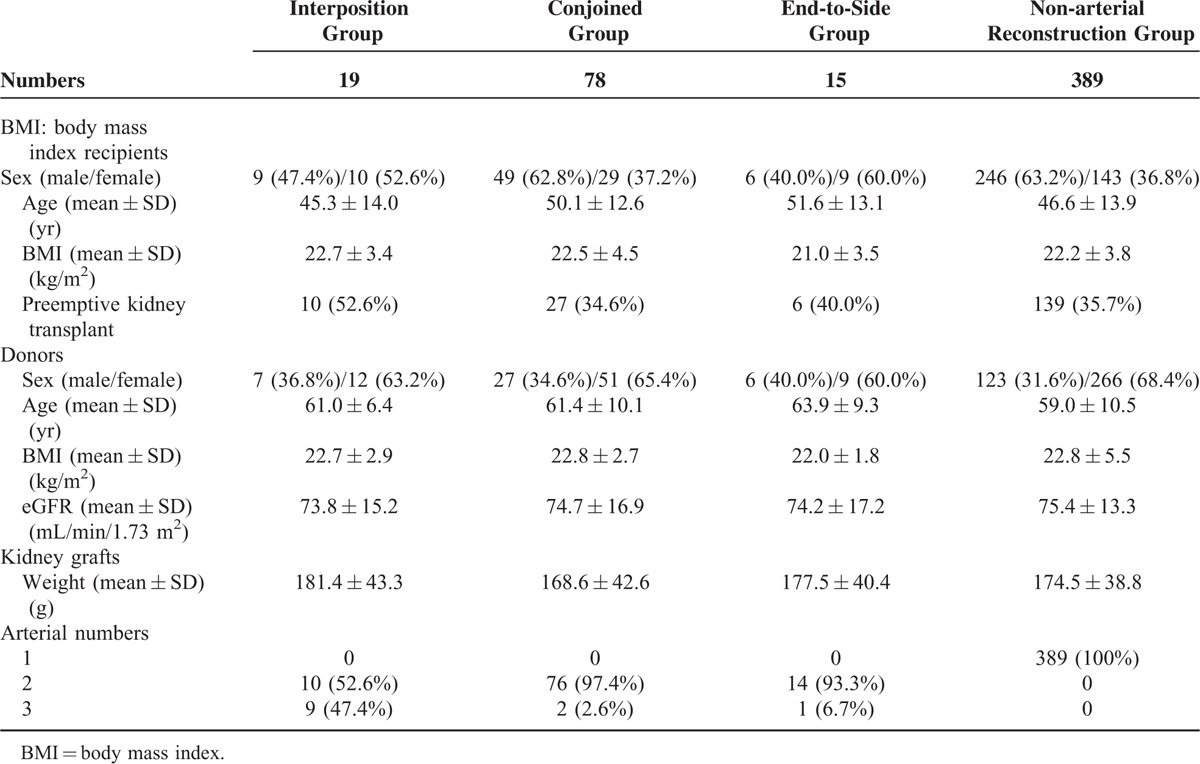
Characteristics of Recipients, Donors, and Kidney Grafts

**TABLE 2:**
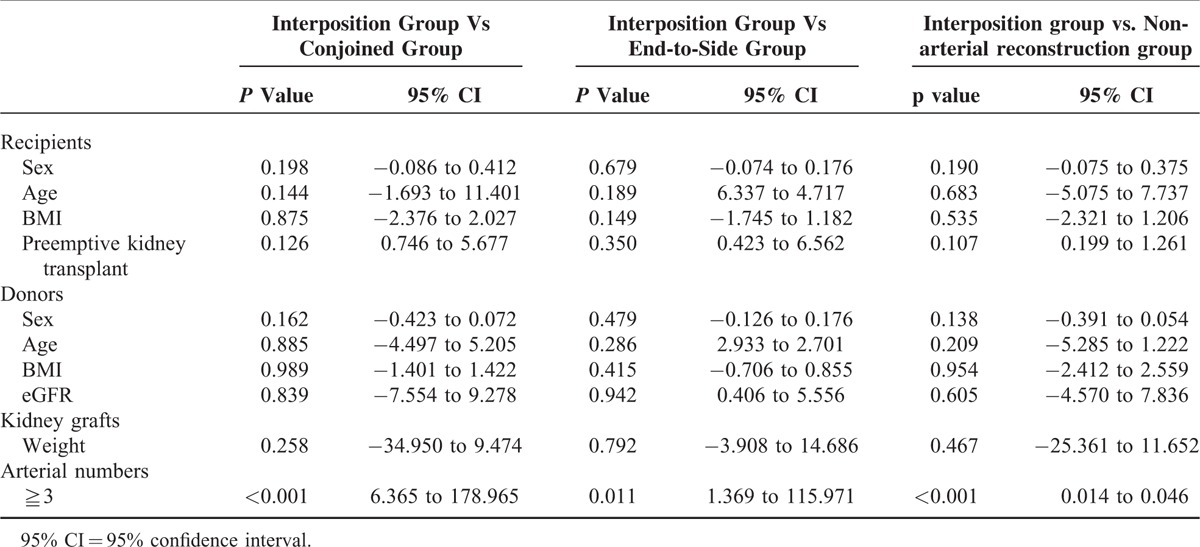
Characteristics of Recipients, Donors, and Kidney Grafts

### Main Results

**FIGURE 3:**
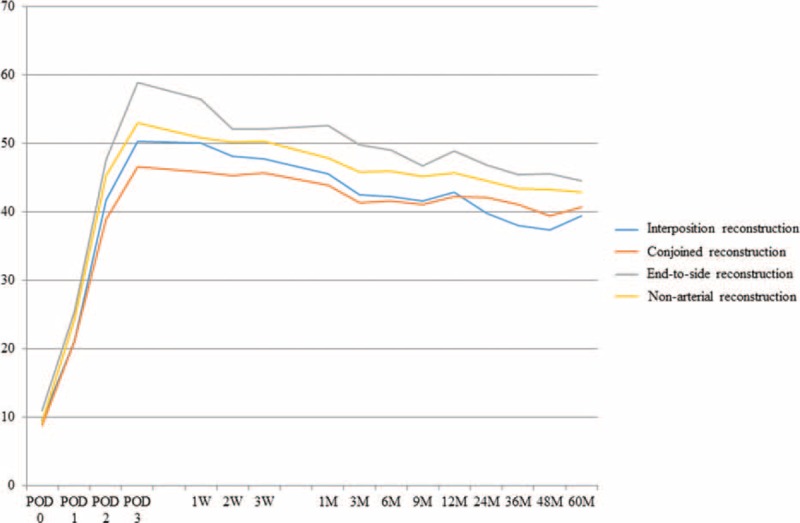
Mean estimated glomerular filtration rate of the recipients during the observation period. There was no significant difference between the 2 groups throughout the perioperative and postoperative periods.

The results of the surgeries were investigated. Warm ischemic time (WIT) (the period between arterial clamp and beginning of the cold perfusion), TIT, time to initial urination and complications were shown in Table 3. WIT in interposition group was significantly longer than that of nonarterial reconstruction group. TIT in interposition group was also significantly longer than those of other 3 groups. However, there was no significant difference in each complication (Table 4). Perioperative and postoperative graft function in interposition group was similar to those of other groups (Fig. 3, Table 5).

**TABLE 3:**
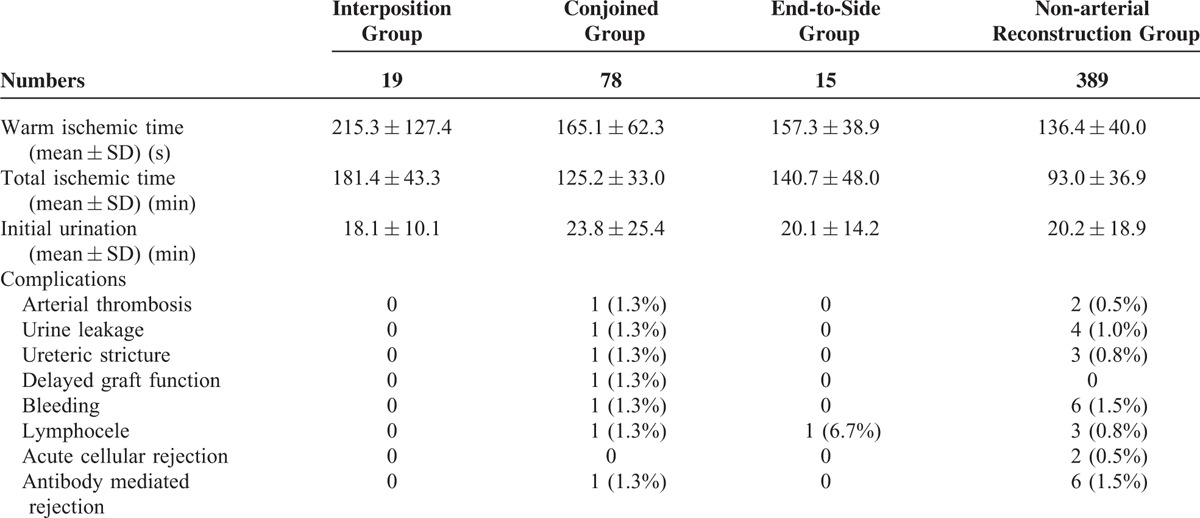
Outcomes of Operation

**TABLE 4:**
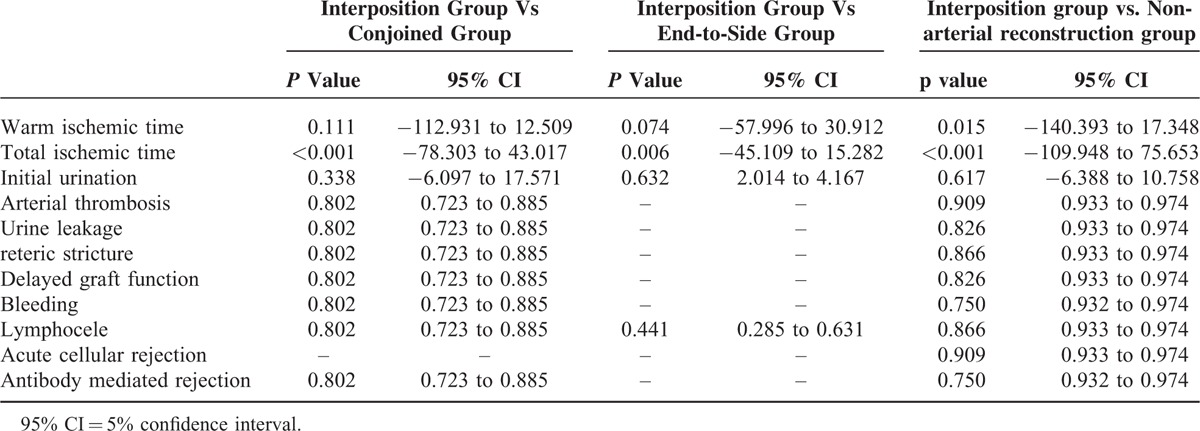
Outcomes of Operation

**TABLE 5:**
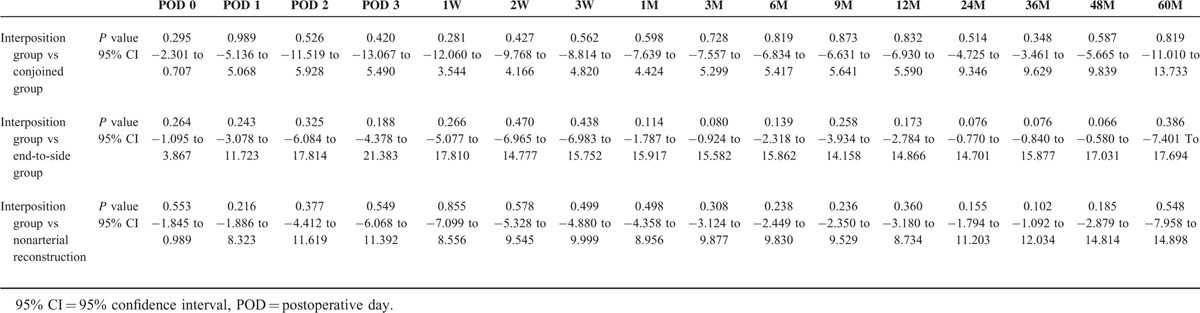
Perioperative and Postoperative Graft Function

## DISCUSSION

It has been reported that more than 2 arteries are found in the unilateral kidneys of 18% to 30% of the potential donors and in the bilateral kidneys of 15% of the potential donors.^13^ Laparoscopic donor nephrectomy has recently been common, but it sometimes leads to short arteries and more than 2 arteries. This might be due to the wide endostaple lines and angle limitation of the endostapler. For these grafts, arterial reconstructions are necessary before transplantation. For the grafts with renal arterial aneurysms, resections of aneurysms, and arterial reconstructions are also required.^14–16^ Reconstructions of accessory arteries to the lower pole are reported to be important to avoid ureteral complications.^17^

Some studies have noted inferior outcomes in multiple graft arterial reconstructions compared with those in a single graft artery.^3,4,14^ Ureteral complications and vascular complications were significantly higher in recipients of multiple arterial grafts. Likewise, lymphoceles were more frequently reported in the recipients of multiple arterial grafts.^18^ Graft survival in recipients of a single arterial graft was significantly better.^4^

On the other hand, several reports have noted the safety and efficacy of arterial reconstruction for multiple arteries.^11,19,20^ In these reports, arterial reconstructions for multiple arteries were performed with multiple end-to-side anastomoses to the external iliac artery, conjoined anastomosis, and end-to-side anastomosis to the main branch of the graft artery.^19^ Outcomes of graft function, graft survival, and complications were similar in recipients of either single or multiple arterial grafts.^3,5,19,21^ However, even in these reports, the interposition method was not discussed closely. Only operative techniques of hypogastric arterial reconstruction and interposition method have been discussed in some reports,^9–12^ but there has been no reports demonstrating the usefulness of the interposition method.

For a successful interposition procedure, preoperative evaluations in both donors and recipients are necessary. Initially, the number of arteries of kidney graft was evaluated with enhanced 3D-CT images. For recipients of multiple kidney graft arteries, CT images of the recipients’ internal iliac artery should be also closely evaluated. Interposition method can be selected for the recipients without calcified internal iliac arteries, because calcification of an internal iliac artery might inhibit the safe of anastomosis and lead to arterial stenosis in the future. For recipients with a calcified internal iliac artery, other procedures such as multiple end-to-side anastomoses to the recipients’ external iliac arteries should be selected.

Characteristics of recipients, donors, and kidney grafts were similar between the interposition group and the other groups except for the arterial number. Interposition method was dominantly selected for the kidney grafts with more than 3 arteries. This meant that interposition method was more applicable in kidney grafts with more than 2 arteries than conjoined method and end-to-side method. And similar outcomes of operative factors, including complications could support this fact. Although arterial thrombosis, ureteric complications, and lymphocele were reported to be more frequent in the multiple arterial reconstruction group, in the present study, these complications did not significantly increase in interposition group compared with conjoined, end-to-side and nonarterial reconstruction groups. WIT in interposition group was similar to conjoined and end-to-side group, but significantly longer than nonarterial reconstruction group. It was because the endostapling of multiple arteries took more time. TIT in interposition group was more time consuming than those in the other 3 groups because of multiple anastomoses in bench surgery. However, these longer WIT and TIT did not affect early graft function, which was evaluated by initial urination, delayed graft function, and perioperative eGFR. In addition, long-term outcome of eGFR in the interposition group was similar not only to those of the conjoined group and end-to-side group but also to that of the nonarterial reconstruction group. This fact was essential to confirm the stability of interposition method. The similar characteristics of recipients, donors, and kidney grafts among these groups strongly supported the rationale for the evaluation of graft function with perioperative and postoperative eGFR. These favorable results were because of the safe and easy technique of interposition method. Each graft artery was anastomosed end-to-end with each branch of the recipient's iliac artery, and bench surgery is conducive to performing these fine procedures. Re-anastomosis of the graft iliac artery with the graft iliac artery was also quite easy because the size of the graft iliac artery was the same.

One limitation of this method is the condition of the recipient's iliac artery. Severely calcified iliac arteries prevent this method and require the selection of a different approach. Another limitation is that the number of interposition group was not large in this study and further prospective investigations are expected.

In conclusion, arterial reconstruction with the recipient's internal iliac artery of multiple kidney graft arteries is a useful standard method for multiple kidney graft arteries of living donor kidney transplantation in carefully selected recipients without calcification of the iliac arteries.

## References

[1] Ratner LE Ciseck LJ Moore RG Laparoscopic live donor nephrectomy. *Transplantation* 1995; 60:1047–1049. 7491680

[2] Wolf JJ Tchetgen M Merion R Hand-assisted laparoscopic live donor nephrectomy. *Urology* 1998; 52:885–887. 9801121 10.1016/s0090-4295(98)00389-6

[3] Carter JT Freise CE McTaggart RA Laparoscopic procurement of kidneys with multiple renal arteries is associated with increased ureteral complications in the recipients. *Am J Transplant* 2005; 5:1312–1318. 15888035 10.1111/j.1600-6143.2005.00859.x

[4] Paramesh A Zhang R Florman S Laparoscopic procurement of single versus multiple artery kidney allograft: is long-term graft survival affected? *Transplantation* 2009; 27:1203–1207. 19935374 10.1097/TP.0b013e3181ba343a PMC2872244

[5] Desai MR Ganpule AP Gupta R Outcome of renal transplantation with multiple versus single renal arteries after laparoscopic live donor nephrectomy: a comparative study. *Urology* 2007; 5:824–827. 17482914 10.1016/j.urology.2007.01.026

[6] Hsu TH Su Li Ratner LE Impact of renal artery multiplicity on outcome of renal donors and recipients in laparoscopic donor nephrectomy. *Urology* 2003; 61:323–327. 12597939 10.1016/s0090-4295(02)02124-6

[7] Troppmann C Wiesmann K McVicar JP Increased transplantation of kidneys with multiple renal arteries in the laparoscopic live donor nephrectomy era: surgical technique and surgical and nonsurgical donor and recipient outcomes. *Arch Surg* 2001; 136:897–907. 11485525 10.1001/archsurg.136.8.897

[8] Antonopoulos IM Yamacake KG Oliveira LM Revascularization of living-donor kidney transplant with multiple arteries: long-term outcomes using the inferior epigastric artery. *Urology* 2014; 84:955–959. 25135869 10.1016/j.urology.2014.06.022

[9] Firmin LC Johari Y Nicholson ML Explantation of the recipient internal iliac artery for bench-surgery during live donor renal transplants with multiple renal arteries. *Ann R Coll Surg Engl* 2010; 92:356. 20514719 10.1308/003588410X12664192076458d PMC3025215

[10] Tchervenkov JI Munda R The use of the hypogastric arteric in the anastomosis of multiple renal arteries in the transplant patient. *Transplant Int* 1990; 3:116–117. 10.1007/BF00336216 2206217

[11] Makiyama K Tanabe K Ishida H Successful renovascular reconstruction for renal allografts with multiple renal arteries. *Transplantation* 2003; 75:828–832. 12660510 10.1097/01.TP.0000054461.57565.18

[12] Pan G Chen Z Liao D The application of the iliac artery in the ex vivo reconstruction of renal arteries in renal transplantation. *Transplantation* 2010; 89:1113–1116. 20179665 10.1097/TP.0b013e3181d54b8e

[13] Roza AM Perloff LJ Naji A Living related donors with multiple renal arteries: a twenty years experience. *Transplantation* 1989; 47:397–399. 2645725

[14] Kadotani Y Okamoto M Akioka K Management and outcome of living kidney grafts with multiple arteries. *Surg Today* 2005; 35:459–466. 15912293 10.1007/s00595-004-2967-2

[15] Jung CW Park KT Kim MG Experiences of renal transplantations from donors with a renal artery aneurysm after a laparoscopic donor nephrectomy and ex vivo reconstruction of renal artery. *Exp Clin Transplant* 2013; 11:324–326. 23905911 10.6002/ect.2013.0023

[16] Toda F Tanabe K Ishikawa N Kidney transplantation from living donors with renal artery disease. *Transplant Proc* 2000; 32:1591–1592. 11119850 10.1016/s0041-1345(00)01344-0

[17] Kok NF Dols LF Hunink MG Complex vascular anatomy in live kidney donation: imaging and consequences for clinical outcome. *Transplantation* 2008; 85:1760–1765. 18580468 10.1097/TP.0b013e318172802d

[18] Mazzucchi E Souza AA Nahas WC Surgical complications after renal transplantation in grafts with multiple arteries. *Int Braz J Urol* 2005; 31:125–130. 15877831 10.1590/s1677-55382005000200006

[19] Benedetti E Troppmann C Gillingham K Short and long term outcomes of kidney transplants with multiple renal arteries. *Ann Surg* 1995; 221:406–414. 7726677 10.1097/00000658-199504000-00012 PMC1234591

[20] Oh HK Hawasli A Cousins G Management of renal allografts with multiple renal arteries resulting from laparoscopic living donor nephrectomy. *Clin Transplant* 2003; 17:353–357. 12868992 10.1034/j.1399-0012.2003.00058.x

[21] Basaran O Moray G Emiroglu R Graft and patient outcome among recipients of renal grafts with multiple arteries. *Transplant Proc* 2004; 36:102–104. 15013313 10.1016/j.transproceed.2003.11.012

